# Factors Associated with Maternal Morbidity in Patients with Eclampsia in Three Obstetric Intensive Care Units: A Retrospective Study

**DOI:** 10.3390/jcm13216384

**Published:** 2024-10-25

**Authors:** Carolina Susanu, Ingrid-Andrada Vasilache, Anamaria Harabor, Petronela Vicoveanu, Alina-Mihaela Călin

**Affiliations:** 1 Clinical and Surgical Department, Faculty of Medicine and Pharmacy, “Dunarea de Jos” University, 800008 Galati, Romania; carolinasusanu@yahoo.com (C.S.); ana.harabor@ugal.ro (A.H.); alina_calin_2005@yahoo.com (A.-M.C.); 2 Department of Mother and Child Care, “Grigore T. Popa” University of Medicine and Pharmacy, 700115 Iasi, Romania; 3 Department of Mother and Newborn Care, Faculty of Medicine and Biological Sciences, “Ștefan cel Mare” University, 720229 Suceava, Romania; petronela.vicoveanu@usm.ro

**Keywords:** eclampsia, comorbidities, risk factors, postpartum

## Abstract

**(1) Introduction**. Eclampsia is a rare complication that can occur during pregnancy and has a significant impact on maternal and neonatal outcomes. The aim of this study was to investigate the risk factors associated with significant maternal morbidity after an eclamptic seizure. **(2) Methods.** An observational retrospective study was performed in three maternity hospitals in Romania between 2015 and 2023 and included pregnant patients diagnosed with eclampsia. Clinical and paraclinical data were investigated, and the impact of several risk factors was assessed using multiple logistic regression analysis. The results were reported as risk ratios (RRs) and 95% confidence intervals (Cis). **(3) Results.** A total of 104 patients with preeclampsia, of whom 23 experienced eclamptic seizures, were included in this study. A total of 82.6% of the patients diagnosed with eclampsia experienced a form of significant morbidity (stroke, PRES syndrome, or any organ failure/dysfunction). Our regression analysis revealed that advanced maternal age (RR: 12.24 95% CI: 4.29–36.61, *p* = 0.002), the presence of thrombotic disorders (RR: 9.17, 95% CI: 3.41–23.70, *p* = 0.03), obesity (RR: 4.89, 95% CI: 0.78–18.15, *p* = 0.036), and smoking status (RR: 2.18, 95% CI: 0.13- 6.51, *p* = 0.042) significantly increase the risk of maternal comorbidities. **(4) Conclusions.** Careful monitoring of pregnant patients, adequate weight control during pregnancy, and correct anticoagulation of individual patients could reduce the extent of postpartum comorbidities that can result from an eclamptic seizure.

## 1. Introduction

Eclampsia is a critical hypertensive disorder in pregnancy that poses significant risks to both maternal and neonatal health outcomes. Despite advancements in maternal healthcare, eclampsia continues to be a major cause of morbidity and mortality globally, particularly in low-resource settings where access to prenatal care and timely intervention remains limited. The pathophysiology of eclampsia, while not fully understood, is believed to involve a complex interplay of endothelial dysfunction, increased blood–brain barrier permeability, and abnormal vascular reactivity [1]. These factors contribute to the development of seizures and other severe complications, necessitating comprehensive management strategies that include early detection, prophylactic measures, and intensive monitoring.

The global burden of eclampsia underscores the need for enhanced screening and prevention efforts, particularly in populations at higher risk for hypertensive disorders of pregnancy. Current research highlights the importance of first-trimester screening for preeclampsia risk factors and the early initiation of prophylactic interventions, such as low-dose aspirin, to mitigate the potential for adverse outcomes [2,3,4].

Eclampsia remains a leading cause of maternal mortality, responsible for the deaths of over 50,000 women annually [5]. The incidence of eclampsia varies significantly between regions, with reported rates of 1.6–10 per 10,000 births in developed countries compared to 50–151 per 10,000 births in developing countries [6]. Moreover, maternal and perinatal mortality and morbidity rates are substantially higher in low-resource settings [7]. This disparity in incidence and pregnancy outcomes is likely attributable to differences in access to prenatal care, early detection of preeclampsia, timely delivery, and the availability of healthcare resources between developed and developing nations.

Eclampsia is characterized by tonic–clonic or multifocal seizures in a woman with preeclampsia, in the absence of epilepsy, ischemia, intracranial hemorrhage, or the influence of drugs. These seizures typically last between 60 and 75 s and can induce fetal distress and bradycardia [8]. Notably, many maternal deaths associated with hypertension are preventable and treatable [9]. The condition is multifactorial, necessitating preventive measures that focus on diet, lifestyle, family history, and obstetric history [9,10].

The management of pregnant women with eclampsia requires intensive care unit (ICU) admission, where comprehensive monitoring is feasible. Key parameters to be monitored in these patients include heart rate, ventilation, and gas exchange, as well as the potential development of HELLP syndrome, which is sometimes associated with eclampsia.

The pathogenesis of eclamptic seizures remains incompletely understood; however, the leading hypothesis suggests that disruption of the blood–brain barrier (BBB), allowing the passage of plasma fluids, ions, and proteins into the brain parenchyma, plays a central role [11,12]. Evidence indicates generalized endothelial cell dysfunction accompanied by abnormal vascular reactivity [8] also plays a role. Recent studies propose that increased blood–brain barrier permeability may be influenced by circulating factors present in the plasma of preeclamptic women, including vascular endothelial growth factor and placental growth factor [13].

Management of eclamptic seizures typically involves supportive care to prevent severe maternal injury, administration of magnesium sulfate to prevent seizure recurrence, oxygen therapy, maintenance of a clear airway, and the facilitation of labor [14,15]. Although routine imaging is not recommended following an eclamptic seizure, the classical radiological finding associated with this condition is posterior reversible encephalopathy syndrome (PRES) [16,17].

Eclampsia is associated with a heightened risk of maternal morbidity, including complications such as abruptio placentae, disseminated intravascular coagulation, pulmonary edema, aspiration pneumonia, cardiopulmonary arrest, and acute renal failure [18,19,20,21]. Furthermore, a history of eclamptic seizures may be linked to long-term cardiovascular risks and cognitive impairments, particularly memory and concentration difficulties, persisting for years after pregnancy [22,23,24].

Eclampsia represents a significant obstetric emergency that requires immediate and effective intervention to prevent severe maternal and neonatal outcomes. The global variation in the incidence and outcomes of eclampsia underscores the importance of understanding the local context and healthcare infrastructure in managing this condition. In high-resource settings, access to comprehensive prenatal care and advanced medical interventions has led to a decline in eclampsia-related mortality. However, in low-resource settings, where access to such care is limited, the mortality and morbidity associated with eclampsia remain alarmingly high. This disparity highlights the critical need for targeted strategies that address the gaps in prenatal care, early detection, and timely management of eclampsia, particularly in regions with limited healthcare resources. The present study aims to contribute to this understanding by exploring the specific risk factors and outcomes associated with eclampsia in a Romanian cohort of patients, providing insights that could inform both local and global healthcare practices.

## 2. Materials and Methodology

This study was conducted across three tertiary care hospitals in Romania: “Sfântul Apostol Andrei” Emergency County Clinical Hospital in Galati, “Cuza Vodă” Obstetrics and Gynecology Clinical Hospital in Iasi, and “Sfântul Ioan cel Nou” County Emergency Clinical Hospital in Suceava. The hospitals were selected for their high patient volume and capacity to manage severe obstetric complications, including eclampsia. The study population consisted of pregnant women diagnosed with preeclampsia or eclampsia between January 2015 and December 2023.

The inclusion criteria required a confirmed diagnosis of preeclampsia and/or eclampsia, pregnancies reaching at least 24 weeks of gestation or a minimum birth weight of 500 g, maternal age of 18 years or older, and complete medical records. Exclusion criteria included eclamptic crises occurring before 24 weeks of gestation, patients under 18 years old, convulsive crises of non-eclamptic origin (e.g., toxic, post-traumatic), and incomplete medical data.

Clinical and demographic data were retrospectively collected from hospital records, including maternal age, gestational age at diagnosis, parity, body mass index (BMI), smoking status, presence of comorbidities (e.g., hypertension, diabetes, systemic lupus erythematosus, thrombophilia), and obstetric history. Data on the management of eclampsia, including the administration of magnesium sulfate, antihypertensive treatment, and timing and mode of delivery, were also recorded. Outcomes of interest included significant maternal morbidity, such as disseminated intravascular coagulation, acute renal failure, HELLP syndrome, and posterior reversible encephalopathy syndrome (PRES). Neonatal outcomes, including birth weight, Apgar scores, and incidence of neonatal complications, were also documented.

Data from a total of 104 pregnant patients diagnosed with preeclampsia were further analyzed in this study. Preeclampsia without severe features was documented in 81 cases, which constituted group 1, while an eclampsia diagnosis, which corresponds to preeclampsia with severe features, was established for the 23 cases that were included in group 2.

The collected data were analyzed using SPSS version 25.0 (IBM Corp., Armonk, NY, USA). Continuous variables were presented as means with standard deviations, while categorical variables were expressed as frequencies and percentages.

The primary outcome, significant maternal morbidity, was defined as the occurrence of one or more severe complications (e.g., disseminated intravascular coagulation, acute pulmonary edema, HELLP syndrome, PRES). Univariate analysis was initially performed to identify potential risk factors for significant maternal morbidity. Variables that showed a *p*-value of less than 0.05 in the univariate analysis were included in a multinomial logistic regression model to adjust for potential confounders and to assess the independent effect of each risk factor. The results of the regression analysis were reported as risk ratios (RR) with 95% confidence intervals (CI). A *p*-value of less than 0.05 was considered statistically significant.

Also, we performed a logistic regression analysis to evaluate the association between neonatal morbidity (birthweight < 2500 g, Apgar score at birth < 7, the presence of respiratory distress syndrome, and admission to the neonatal intensive care unit (NICU)) and the diagnosis of eclampsia, adjusted for gestational age at birth. The results were reported as adjusted odds ratios (aORs) and 95% confidence intervals (CIs).

The study was conducted in accordance with the Declaration of Helsinki and was approved by the ethics committees of all three participating hospitals. Given the retrospective nature of the study, patient consent was not required; however, all patient data were anonymized to ensure confidentiality. The study’s design and data handling protocols adhered to national and international guidelines for research involving human subjects.

## 3. Results

A total of 104 patients were hospitalized in intensive care units during the study period with preeclampsia (with or without severe features) before delivery. Of these, 23 patients met the inclusion criteria and developed eclamptic seizures (Table 1). Among these patients, 82.6% experienced significant morbidity, in contrast to 16.3% of patients diagnosed with preeclampsia.

The mean age of patients who developed eclampsia was 28.48 years old, compared to 29.92 years old in the preeclampsia with non-severe features group. The distribution of living environments was comparable between both groups. Notably, two (8.6%) of the patients with eclampsia were smokers. Nulliparity was present in 69.5% of the eclampsia group, compared to 48.8% in the preeclampsia group, with a statistically significant difference (*p* = 0.021).

**Figure 1:**
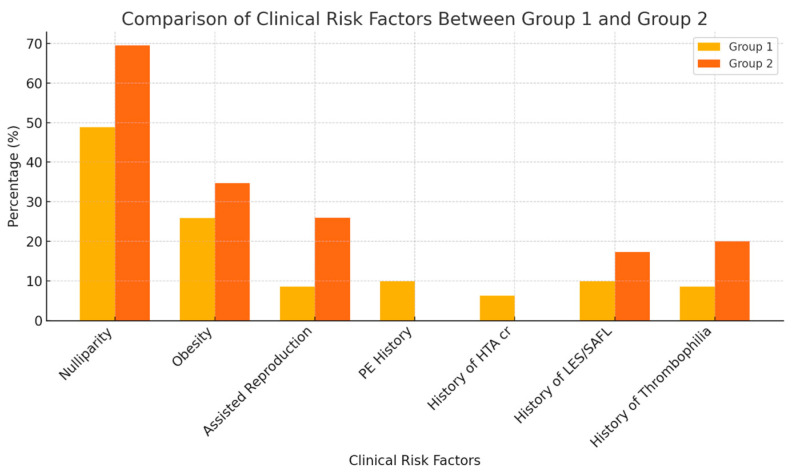
Comparison of clinical risk factors between group 1 and group 2.

Obesity was more prevalent in the eclampsia group (34.7%) than in the preeclampsia group (25.9%). Our results showed that patients who developed eclampsia had significantly higher rates of conception via in vitro fertilization in comparison with patients included in the first group (26% in the eclampsia group versus 8.6% in the preeclampsia group, *p* = 0.007). This descriptive data is presented in Figure 1.

A personal history of preeclampsia (9.9%) and chronic hypertension (6.2%) was documented in the preeclamptic group, but not in the eclampsia group. However, systemic lupus erythematosus was encountered in 17.3% of patients who developed eclamptic seizures, showing a statistically significant difference compared to the preeclamptic group (*p* = 0.004). Additionally, a history of thrombophilia was observed in 20% of eclampsia cases, compared to 8.6% in the preeclampsia cases.

Univariate analysis of the clinical characteristics, as presented in Table 1, revealed that patients who developed eclamptic seizures had higher rates of nulliparity, obesity, and conception via assisted reproductive techniques. These patients also had a significantly more frequent history of systemic lupus erythematosus and thrombophilia (*p* = 0.007).

**Figure 2:**
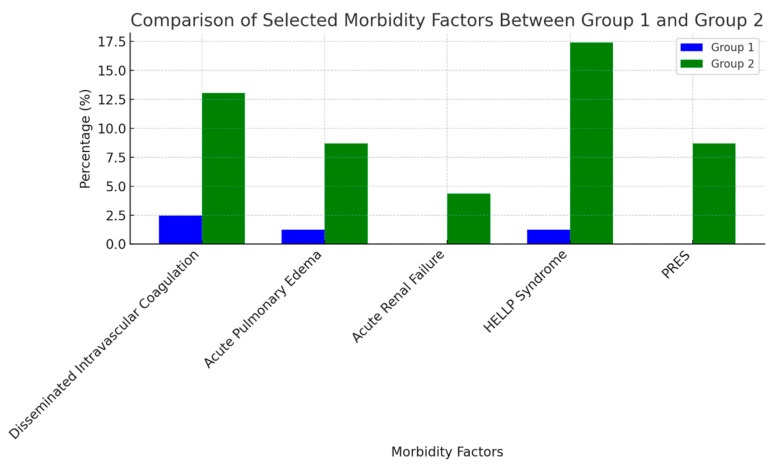
Comparison of selected morbidity factors between group 1 and group 2.

Maternal morbidity rates, as detailed in Table 2, were stratified according to the study groups. Among the eclamptic patients, 13.04% developed disseminated intravascular coagulation syndrome, compared to 2.46% in the preeclampsia group. Acute pulmonary edema was observed in 8.69% of the eclamptic patients, whereas it occurred in only 1.23% of the preeclampsia group. Acute renal failure was identified in a single case (4.34%) within the eclamptic group. HELLP syndrome was present in 17.39% of eclamptic patients, compared to 1.23% in the preeclampsia group. Posterior reversible encephalopathy syndrome (PRES) was detected exclusively in the eclamptic group, with an incidence of 8.69%. These data are graphically presented in Figure 2.

These findings indicate that patients who developed eclampsia exhibited significantly higher rates of disseminated intravascular coagulation (13.04% versus 2.46%, *p* = 0.006), acute renal injury (4.34% versus 0%, *p* = 0.009), HELLP syndrome (17.39% versus 1.23%, *p* = 0.001), and PRES (8.69% versus 0%, *p* = 0.009) compared to those included in the preeclamptic group.

Subsequently, all types of morbidity were consolidated into a composite variable termed “morbidity,” enabling the identification of clinical risk factors that significantly influence the occurrence of major maternal morbidity. These risk factors are detailed in Table 3. Advanced maternal age was associated with a risk ratio of 12.24 (95% confidence interval CI: 4.29–36.61, *p* = 0.002). Obesity presented a risk ratio of 4.89 (95% CI: 0.78–18.15) for significant maternal morbidity.

A history of preeclampsia was also identified as a significant risk factor, with a risk ratio of 4.16 (95% CI: 1.90–9.12) and a statistically significant *p*-value of 0.0004. Additionally, a history of chronic hypertension emerged as a significant risk factor, with a risk ratio of 3.59 (95% CI: 1.57–8.18, *p* = 0.002). The study further underscored the importance of a history of thrombophilia as a risk factor, with a risk ratio of 9.17 (95% CI: 3.41–23.70).

These findings indicate that advanced maternal age, obesity, a personal history of preeclampsia, chronic hypertension, and thrombophilia significantly elevate the risk of maternal morbidity.

In addition to maternal outcomes, neonatal morbidity was assessed among the offspring of the 23 patients who experienced eclamptic seizures. The mean birth weight of neonates in the eclampsia group was 2150 g, significantly lower compared to 2750 g in the preeclampsia control group (*p* = 0.018). The incidence of preterm birth (defined as delivery before 37 weeks of gestation) was notably higher in the eclampsia group at 47.8%, compared to 19.8% in the control group (*p* = 0.004).

Neonates born to mothers in the eclampsia group also had a higher incidence of low Apgar scores (<7 at 5 min) at 26.1%, compared to 11.1% in the control group (*p* = 0.031). Moreover, complications such as respiratory distress syndrome (RDS) and neonatal intensive care unit (NICU) admission were more frequent in the eclampsia group, occurring in 30.4% and 43.5% of cases, respectively, compared to 12.3% and 17.3% in the control group (*p* = 0.011 and *p* = 0.007, respectively). These findings underscore the significant neonatal morbidity associated with eclampsia, highlighting the critical need for intensive neonatal monitoring and care in these cases.

Table 4 presents the results of a logistic regression that investigated the association between eclampsia and the occurrence of neonatal morbidity adjusted for gestational age at birth.

Our results showed that the presence of eclampsia significantly increased the odds of a low gestational age at birth (aOR: 0.45, 95% CI:0.31–0.65, *p* < 0.001), an Apgar score at birth less than 7 (aOR: 0.54, 95% CI: 0.41–0.70, *p* < 0.001), respiratory distress syndrome (aOR: 0.75, 95% CI:0.62–0.91, *p* = 0.004) and NICU admission (aOR: 0.60, 95% CI: 0.40–0.90, *p* = 0.015).

## 4. Discussion

Eclampsia is associated with a modestly elevated risk of maternal death in developed countries; however, in developing countries, maternal mortality rates can reach up to 7% [25]. Despite significant advancements in healthcare, eclampsia continues to be a major contributor to maternal mortality [26]. A recent cross-sectional study involving 29 countries demonstrated a dramatically increased risk of death among women with eclampsia (aOR: 42.38; 95% CI: 25.14–71.44) compared to those without preeclampsia [27]. Furthermore, the risk of life-threatening central nervous system damage in eclamptic women was up to 60 times higher than in those without preeclampsia [27].

Adverse maternal outcomes and mortality related to preeclampsia are most prevalent among women over 35 years of age, Hispanic and African American women, those at 20–28 weeks of gestation, those with multiple pregnancies, and nulliparous women [28,29,30,31]. Our results indicated that the mean maternal age and standard deviation were 28.48 ± 6.76 years in the eclampsia group. Moreover, about 69% of the pregnant patients who developed eclampsia were nulliparous. In a retrospective analysis of preeclampsia-related deaths identified by the California Pregnancy-Associated Mortality Review from 2002 to 2007, 54 (16%) cases were associated with preeclampsia, while eclampsia was reported in 36% of cases [32]. In our study no maternal death was recorded.

In addition to the increased risk of mortality, eclampsia is linked to severe maternal complications, including abruptio placentae, HELLP syndrome, disseminated intravascular coagulation, pulmonary edema, aspiration pneumonia, cardiopulmonary arrest, and acute renal failure [33]. In our study, patients with eclampsia had relatively high rates of disseminated intravascular coagulation (13.04%), acute renal injury (4.34%), HELLP syndrome (17.39%), and PRES (8.69%). Women who develop eclampsia at ≤32 weeks of gestation have a higher incidence of abruptio placentae, HELLP syndrome, and acute renal failure compared to those who develop eclampsia later in pregnancy [34,35,36,37].

Perinatal mortality and morbidity remain significant in eclamptic pregnancies, with reported perinatal death rates ranging from 5.6% to 11.8% [38,39]. Most perinatal deaths are attributed to abruptio placentae, fetal growth restriction, or extreme prematurity. Newborns of women with eclampsia are at increased risk of being small for gestational age (SGA) and experiencing complications related to prematurity, such as respiratory distress syndrome and neonatal death [3,40].

Our results showed that the presence of eclampsia significantly increased the odds of a low gestational age at birth, an Apgar score at birth less than 7, respiratory distress syndrome and NICU admission. Only three neonatal deaths were recorded: two in the non-severe preeclampsia group and one in the eclampsia group.

This study corroborated the elevated rates of maternal morbidity among patients with eclampsia and identified clinical risk factors that significantly influence the occurrence of severe complications. Early prenatal screening, particularly in the first trimester, for the risk of developing preeclampsia, coupled with the initiation of aspirin prophylaxis at a dose of 150 mg/day before 16 weeks of gestation, can substantially reduce the incidence of associated maternal and neonatal complications [41]. Additionally, rigorous monitoring of patients and the implementation of a therapeutic regimen that effectively controls blood pressure may delay the need for fetal delivery and minimize complications associated with prematurity.

However, the study has several limitations, including its retrospective design, the relatively small sample size of patients who developed eclamptic seizures, the absence of long-term follow-up data for these patients, the absence of control for regional disparities, limited neonatal data and lack of generalizability. Nonetheless, the study’s strengths lie in its multi-center approach, conducted across three intensive care units in three distinct clinics.

The analysis of the clinical and demographic data collected from the study population revealed several key findings that underscore the severity and complexity of eclampsia as a maternal health issue. The results are presented in terms of maternal and neonatal outcomes, with a particular focus on the prevalence of significant maternal morbidity and the associated risk factors. The following sections detail the incidence of various complications observed in eclamptic patients, comparing them with the control group diagnosed with preeclampsia. The statistical analysis highlights the critical variables that significantly contribute to adverse maternal and neonatal outcomes, providing a comprehensive overview of the impact of eclampsia within the studied cohort.

The findings of this study emphasize the substantial maternal and neonatal morbidity associated with eclampsia, reinforcing the need for early identification and intervention in at-risk populations. The significantly higher rates of severe complications such as disseminated intravascular coagulation, HELLP syndrome, and posterior reversible encephalopathy syndrome (PRES) in eclamptic patients highlight the critical nature of this condition. Our results are consistent with previous studies that have demonstrated the disproportionate burden of morbidity in patients with eclampsia compared to those with preeclampsia alone. The strong association between advanced maternal age, obesity, and a history of hypertensive disorders with increased morbidity underscores the importance of targeted prenatal care and risk factor management in reducing adverse outcomes.

Thus, clinicians should carefully screen pregnant patients as soon as the first trimester for pregnancy for maternal risk factors for preeclampsia, should provide aspirin prophylaxis (150 mg/day) before 16 weeks of gestation, and should provide intensive care management of those patients with eclamptic seizures. Moreover, magnesium sulphate, which has limited costs, is essential for the prophylaxis of primary and recurrent eclamptic seizures and should be administered under careful medical surveillance for this category of patients. The maternal mortality rate in this study was 0%, mainly due to prompt admission of these patients to tertiary units, which provide advanced medical resources and intensive care. Thus, it is imperative for other low-resource centers to develop dedicated centers that would ultimately greatly reduce maternal and fetal mortality and morbidity rates.

Furthermore, the observed neonatal outcomes in the eclampsia group, including lower birth weights, higher rates of preterm birth, and increased incidences of neonatal intensive care unit admissions, align with the literature documenting the impact of eclampsia on neonatal health [42,43,44]. These findings suggest that the perinatal environment in eclamptic pregnancies is significantly compromised, likely due to factors such as uteroplacental insufficiency and preterm deliveries precipitated by maternal seizures. This study advocates for the implementation of comprehensive antenatal screening programs, particularly in populations with known risk factors, to identify and manage potential complications early on. Additionally, the necessity for multidisciplinary care involving obstetricians, neonatologists, and critical care specialists is evident to optimize outcomes for both the mother and the neonate in cases of eclampsia [45].

Given the study’s retrospective design and the relatively small sample size, further research is warranted to confirm these findings and explore additional preventive and therapeutic strategies. Future studies should also focus on long-term follow-up to better understand the chronic sequelae of eclampsia on maternal and neonatal health.

First trimester screening for preeclampsia and aspirin prophylaxis are two essential steps required for greatly reducing the incidence of maternal and neonatal complications, and further studies should focus on identifying the best predictive markers and screening strategies that would allow for better risk stratification of pregnant patients.

Ultimately, this study calls for continued efforts to improve the identification, prevention, and management of eclampsia, particularly in resource-limited settings where the burden of this condition is most profound. By addressing these challenges, healthcare providers can significantly reduce the incidence of severe morbidity and improve overall maternal and neonatal outcomes.

## 5. Conclusions

This study underscores the significant maternal and neonatal morbidity associated with eclampsia, a condition that remains a critical challenge in obstetric care despite advances in maternal health management.

The data reveal that eclampsia is not only a leading cause of severe maternal complications, such as disseminated intravascular coagulation, HELLP syndrome, and posterior reversible encephalopathy syndrome, but also that it significantly compromises neonatal outcomes, leading to higher rates of preterm birth, low birth weight, and increased neonatal intensive care unit admissions.

The identification of key clinical risk factors—such as advanced maternal age, obesity, and a history of hypertensive disorders—provides valuable insights for the early detection and management of preeclampsia and eclampsia.

These findings suggest that enhanced prenatal screening, beginning as early as the first trimester, and the early implementation of prophylactic interventions, like low-dose aspirin, can play a crucial role in mitigating the risks associated with these conditions.

Furthermore, the study highlights the importance of individualized and multidisciplinary care approaches, which are essential in managing the complex interplay of maternal and fetal health in eclampsia cases.

## Figures and Tables

**Table 1:** Clinical characteristics of the patients included in the study.

Maternal Characteristics	Group 1 (Preeclampsia, *n* = 81 Patients)	Group 2 (Eclampsia, *n* = 23 Patients)	*p* Value
Age, years (mean ± SD)	29.92 ± 6.92	28.48 ± 6.76	0.18
Living environment (*n*/%)	Urban = 33 (40.7%) Rural = 48 (59.3%)	Urban = 9 (39.1%) Rural = 47 (60.8%)	0.94
Parity (*n*/%)	Nulliparous = 39 (48.8%) Multiparous = 42 (51.2%)	Nulliparous = 16 (69.5%) Multiparous = 7 (30.4%)	0.021
Obesity (*n*/%)	Yes = 21 (25.9%)	Yes = 8 (34.7%)	<0.001
IVF conception (*n*/%)	Yes = 7 (8.6%)	Yes = 6 (26%)	0.007
Smoking (*n*/%)	Yes = 0 (0%)	Yes = 2 (8.6%)	0.15
History of PE (*n*/%)	Yes = 8 (9.9%)	Yes = 0 (0%)	0.004
History of chronic hypertension (*n*/%)	Yes = 5 (6.2%)	Yes = 0 (0%)	0.024
History of chronic kidney disease (*n*/%)	Yes = 1 (1.2%)	Yes = 0 (0%)	0.31
History of diabetes mellitus (*n*/%)	Da = 1 (1.2%)	Da = 0 (0%)	0.31
History of SLE/APS (*n*/%)	Yes = 8 (9.9%)	Yes = 4 (17.3%)	0.004
History of thrombophilia (*n*/%)	Yes = 7 (8.6%)	Yes = 3 (20%)	0.007
SBP, mmHg (mean ± SD)	167.01 ± 16.59	197.5 ± 10.82	<0.001
DBP, mmHg (mean ± SD)	85.30 ± 7.71	95.12 ± 5.45	<0.001

Legend: SD—standard deviation, IVF—in vitro fertilization, PE—preeclampsia, SLE—systemic lupus erythematosus, APS—antiphospholipid syndrome, SBP—systolic blood pressure, DBP—syastolic blood pressure.

**Table 2:** Maternal morbidity rates segregated by study groups.

Maternal Morbidity	Group 1 (Preeclampsia, *n* = 81 Patients)	Group 2 (Eclampsia, *n* = 23 Patients)	*p* Value
Abruptio placentae (*n*/%)	Yes = 11 (13.58%)	Yes = 5 (21.73%)	0.33
DIC (*n*/%)	Yes = 2 (2.46%)	Yes = 3 (13.04%)	0.006
APE (*n*/%)	Yes = 1 (1.23%)	Yes = 2 (8.69%)	0.05
AKI (*n*/%)	Yes = 0 (0%)	Yes = 1 (4.34%)	0.009
HEELP syndrome (*n*/%)	Yes = 1 (1.23%)	Yes = 4 (17.39%)	0.001
PRES (*n*/%)	Yes = 0 (0%)	Yes = 2 (8.69%)	0.009

Legend: DIC—disseminated intravascular coagulation, APE—acute pulmonary edema, AKI—acute kidney injury, HEELP syndrome—hemolysis, elevated liver enzymes, low platelets count, PRES—posterior reversible encephalopathy syndrome.

**Table 3:** Clinical risk factors for maternal morbidity.

Maternal Clinical Risk Factors	RR and 95% CI	*p* Value
Advanced maternal age	12.24 (4.29–36.61)	0.002
Nulliparity	1.56 (0.31–7.86)	0.58
Obesity	4.89 (1.78–18.15)	0.036
IVF conception	1.26 (0.11–5.33)	0.42
History of PE	4.16 (1.90–9.12)	0.0004
History of chronic hypertension	3.59 (1.57–8.18)	0.002
History of chronic kidney disease	1.42 (0.19–9.42)	0.36
History of diabetes mellitus	0.86 (−0.24–7.81)	0.89
History of SLE/APS	0.57 (0.11–5.33)	0.42
History of thrombophilia	9.17 (3.41–23.70)	0.03

Legend: RR–risk ratio, CI–confidence intervals, IVF–in vitro fertilization, PE–preeclampsia, SLE–systemic lupus erythematosus, APS–antiphospholipid syndrome.

**Table 4:** Logistic regression that evaluated the association between neonatal morbidity and the presence of eclampsia adjusted for gestational age at birth.

Neonatal Outcome	Adjusted Odds Ratio	95% Confidence Interval	*p* Value
Gestational age at birth less than 2500 g	0.45	0.31–0.65	<0.001
Apgar score at birth < 7	0.54	0.41–0.70	<0.001
Respiratory distress syndrome	0.75	0.62–0.91	0.004
NICU admission	0.60	0.40–0.90	0.015

Legend: NICU–neonatal intensive care unit admission.

## Data Availability

The datasets produced and/or analyzed in the present study can be obtained from the corresponding authors upon request made in a reasonable manner.
